# Assessment of cerebrovascular disease with computed tomography in COVID-19 patients: correlation of a novel specific visual score with increased mortality risk

**DOI:** 10.1007/s11547-020-01313-9

**Published:** 2020-11-28

**Authors:** Andrea Bianchi, Lorenzo Nicola Mazzoni, Simone Busoni, Nicola Pinna, Marco Albanesi, Edoardo Cavigli, Diletta Cozzi, Anna Poggesi, Vittorio Miele, Enrico Fainardi, Davide Gadda

**Affiliations:** 1 Department of Neuroradiology, Careggi University Hospital, Florence, Italy; 2 Medical Physics Department, Careggi University Hospital, Florence, Italy; 3 Medical Physics Unit, AUSL Toscana Centro, Prato, Pistoia Italy; 4 Department of Clinical and Experimental Medicine, Institute of Diagnostic Imaging 2, University of Sassari, Sassari, Italy; 5 Department of Emergency Radiology, Careggi University Hospital, Largo Brambilla 3, 50134 Florence, Italy; 6 NEUROFARBA Department, Neuroscience Section, University of Florence, Careggi University Hospital, Florence, Italy; 7 Stroke Unit, Careggi University Hospital, Florence, Italy; 8 Department of “Scienze Biomediche, Sperimentali E Cliniche”, Neuroradiology, University of Florence, Careggi University Hospital, Florence, Italy

**Keywords:** Coronavirus disease, CT, Risk factor, Cerebrovascular disease, Mortality

## Abstract

**Purpose:**

Cerebrovascular disease (CVD) is considered a major risk factor for fatal outcome in COVID-19. We aimed to evaluate the possible association between computed tomography (CT) signs of chronic CVD and mortality in infected patients.

**Materials and methods:**

We performed a double-blind retrospective evaluation of the cerebral CT scans of 83 COVID-19 patients looking for CT signs of chronic CVD. We developed a rapid visual score, named CVD-CT, which summarized the possible presence of parietal calcifications and dolichosis, with or without ectasia, of intracranial arteries, areas of chronic infarction and leukoaraiosis. Statistical analysis was carried out with weighted Cohen’s K test for inter-reader agreement and logistic regression to evaluate the association of in-hospital mortality with CVD-CT, chest X-ray (CXR) severity score (Radiographic Assessment of Lung Edema-RALE) for radiological assessment of pulmonary disease, sex and age.

**Results:**

CVD-CT (odds ratio 1.6, 95% C.I. 1.2-2.1, *p* = 0.001) was associated with increased risk of mortality. RALE showed an almost significant association (odds ratio 1.05, 95% C.I. 1-1.1, p 0.06), whereas age and sex did not.

**Conclusion:**

CVD-CT is associated with risk of mortality in COVID-19 patients. The presence of CT signs of chronic CVD may be correlated to a condition of fragility of the circulatory system, which constitutes a key risk factor for death in infected patients.

## Introduction

Since the first report of the outbreak in Wuhan, China in December 2019, as of August 26, 2020, the World Health Organization has confirmed more than 23.7 million cases of the novel coronavirus (SARS-CoV-2) infectious disease named COVID-19, with more than 815.000 deaths [1, 2]. The ongoing COVID-19 pandemic, the most severe after 1918 influenza, is a worldwide health emergency with major socioeconomic consequences.

Although the common presentation is respiratory disease, there is increasing evidence of a multi-organ involvement of COVID-19, including cardiovascular and central nervous systems [3, 4]. As a matter of fact, angiotensin-converting enzyme 2 (ACE2), which SARS-CoV-2 binds to penetrate the target cells, is widely expressed in various organs and tissues, including the cardiovascular, digestive and urogenital systems [5]. Moreover, it is present in brain vascular endothelium [5].

Identification of prognostic and risk factors for severe evolution is crucial for better understanding of the disease, definition of the most effective treatment strategies and research orientation. The association between severe illness and fatal outcome with advanced age and various comorbidities, including cerebrovascular disease (CVD), has been demonstrated [6, 7].

Computed tomography (CT) may reveal signs of CVD, related to both large and small vessels pathology, i.e., calcifications, elongation and/or ectasia of intracranial arteries, hypodense areas of post-infarction encephalomalacia/gliosis and leukoaraiosis [8–11].

We aimed to develop a rapid visual score summarizing major CT signs of chronic CVD and to investigate its possible correlation with mortality risk in COVID-19 patients.

## Materials and methods

### Patients enrollment

From March 1 to April 17 2020, 398 patients, (161 females; 237 males; mean age 66 ± 16.9 years, range 21–97) evaluated at the emergency department of our University Hospital, had a positive RT-PCR nasopharyngeal–throat swab for COVID-19 infection. Among these, 83 patients (34 females; 49 males; mean age 72 ± 14.8 years, range 26–97) underwent a brain CT scan in the time lapse of 60 days before and 30 days after the diagnosis of COVID infection and they represent the population of our study.

The brain CT scans were performed for different clinical reasons, most frequently disorders of consciousness (47%), head trauma with or without syncope (23%) and balance disorders (9%), and acquired with different scanners using a standard head protocol: sequential acquisition on a Siemens Somatom definition 128-slice scanner and on a GE Optima CT660 64-slice scanner and a multislice helical technique on a Philips ICT Brilliance 128-slice scanner. Reconstructed slice thickness ranged between 2.4 and 3 mm. Images were evaluated utilizing standard brain window (W80-C40).

### Radiographic Assessment of Lung Edema (RALE) score evaluation

The extent of COVID-19 lung involvement was assessed by two thoracic radiologists with a chest X-ray (CXR) severity score (Radiographic Assessment of Lung Edema-RALE) [12]. Following RALE indications, each CXR was evaluated by means of a score between 0 and 48, ranging from the absence of any pathological sign (score 0) to the complete pathological involvement of lung parenchyma (score 48).

### Cerebrovascular disease computed tomography (CVD-CT) index

Each CT examination of the brain was double-blinded evaluated by two neuroradiologists (DG, with 24 and AB, 10 years experience). Each neuroradiologist assigned dichotomous scores in case of the absence or presence of the following signs, which had been related to a possible condition of large vessel disease [8–10]: intracranial arterial calcification (index C), hypodense areas of post-infarction encephalomalacia (index H) and elongation with or without ectasia of at least one major intracranial artery (index D, as dolichosis).

For index H, fronto-basal and temporo-polar areas of encephalomalacia were not considered, due to the most probable post-traumatic origin of such lesions.

For index D, the readers evaluated the vertebro-basilar trunk, since more frequently affected by dolichoectatic conditions as compared to the anterior circle. A vertebral artery (VA) was considered elongated when it approximated the contralateral margin of the clivus or when it was displaced lateral to the ipsilateral margin of the clivus. Basilar artery (BA) was considered elongated if displaced lateral to the clivus or the dorsum sellae or when BA bifurcation approximated the third ventricle floor. BA was ectatic when larger than 4.5 mm [13].

Afterward, the readers assessed the possible leukoaraiosis extent by means of Van Swieten scale. After distinguishing the two most affected regions (around the anterior horns of lateral ventricles and the posterior parts of the cella media and the centrum semiovale), the severity of white matter (WM) lesions was graded as 0, 1 and 2 for each level, with a final score ranging from 0 to 4 [14].

Two new indices were subsequently introduced: large vessel disease (LVD) index and moderate to severe leukoaraiosis (MSL) index. LVD is a dichotomous index which is equal to 1 if 2 out of 3 of the values of the indexes C, H and D were positive for both readers, otherwise equal to 0. MSL is a dichotomous index which is equal to 1 if the Van Swieten score is > 2 for both radiologists, otherwise equal to 0. In order to assess the inter-rater agreement of the dichotomous diagnosis of LVD and MSL achieved by a single reader from the above described conditions (the presence of 2 or 3 LVD parameters and a Van Swieten score > 2), the two indices were also calculated in a "single reader" version (LVDsr and MSLsr, respectively).

Cerebrovascular disease computed tomography (CVD-CT) index is defined by the following equation

1 $${\text{CVD-CT}} = {\text{ a}} \times {\text{LVD}} + {\text{b}} \times {\text{MSL}}$$

where a and b are the weights that consider the association of LVD and MSL with mortality and are estimated using Cohen's constant K. Both are equal to the corresponding value of K multiplied by 10. In this way, a synthetic index is defined that considers the contribution of all the previously introduced indices to evaluate the CT examination of the brain, furthermore weighting LVD and MSL with their association with mortality.

### Statistical analysis

Inter-reader agreement in estimating the C, H, D, Van Swieten, LVDsr and MSLsr indices was assessed through the weighted Cohen’s K test. Differences of sex, age, RALE and CVD-CT among groups of patients with different outcome are established by means of nonparametric Mann–Whitney test; corresponding descriptive statistics is reported.

A forward and backward logistic regression analysis with maximum likelihood method was performed to evaluate the association of patients' treatment (categorized as 0 = medical treatment at home, 1 = hospitalization into a medicine department, 2 = hospitalization into an intensive care unit with noninvasive ventilation, 3 = hospitalization into an intensive care unit with invasive ventilation), RALE and CVD-CT with in-hospital death after positive RT-PCR swab for COVID-19, considering sex and age as covariates. Predictors were recursively inserted or excluded from the regression model in case of significance of the change with *p* < or > 0.05, respectively. Goodness of fit statistics was established by means of Hosmer–Lemeshow test. Odds ratios and corresponding 95% confidence intervals (95% CI) were thus estimated by logistic regression model. Statistical analysis was performed with SPSS (IBM SPSS Statistics for Windows, Version 26.0), statistical significance threshold was set at *p* = 0.05.

## Results

After the diagnosis of COVID-19 infection, no patient of our study group was dismissed from hospital for treatment at home, 46 out of 83 (55%) were admitted into a medicine department and most of the others (35 patients, 42%) received invasive mechanical ventilation.

Twenty-five patients (30%) died during hospitalization, 10 of whom had received invasive mechanical ventilation (28% in this group), whereas 15 had not (32%). Both patients with noninvasive mechanical ventilation survived. The total number of C, H, D, LVDsr and MSLsr indexes identified by both readers among survivors and deceased patients and the weighted Cohen’s K calculated to evaluate inter-reader agreement is reported in Table 1. It can be noticed that the MSLsr, Van Swieten and D indexes showed the best inter-reader agreement, H and LVDsr indexes a moderate-to-good agreement while the C index a poor one. The Cohen’s K values calculated to evaluate the association of the LVD and MSL indices with mortality were 0.3 and 0.2, respectively. Then, the values of parameters a and b, defined in Eq. 1, were set equal to 3 and 2, respectively. CVD-CT index was thus finally obtained for each patient. Descriptive statistics of age, RALE and CVD-CT score divided by survival or death, with corresponding* p* values calculated by Mann–Whitney test, are reported in Table 2. It is noteworthy that only the CVD-CT showed statistically significant differences between the two groups.

**Table 1:** Total number and percentage of cases identified by both readers for each parameter among survivors and deceased patients, weighted Cohen’s K for inter-reader agreement evaluation and percentage of identical scoring

	C	H	D	Van Swieten	LVDsr	MSLsr
Cases (%) for both readers in survivors	36 (62%)	4 (6%)	29 (50%)	non-dichotomous	17 (29%)	5 (8%)
Cases (%) for both readers in deceased	21 (84%)	4 (16%)	14 (56%)	non-dichotomous	17 (68%)	9 (36%)
Cohen’s K	0.34	0.58	0.79	0.78	0.54	0.92
Percentages of identically classified patients	76	88	90	69	77	98

**Table 2:** Descriptive statistics of age, RALE and CVD-CT divided by outcome, with corresponding *p* value after Mann–Whitney test

	Survivors (N = 58; 31 males, 27 females)				Deceased (N = 25; 18 males, 7 females)				*p* value
	Mean	Median	Min	Max	Mean	Median	Min	Max	
Age (years)	70.2	71.5	26	96	76.3	77.0	45	97	0.11
RALE	12.2	9	0	44	15.8	14	0	40	0.13
CVD-CT	1.1	0	0	5	2.7	3	0	5	0.002

*RALE* Radiographic Assessment of Lung Edema score*, CVD-CT* Cerebrovascular disease computed tomography score

**Fig. 1:**
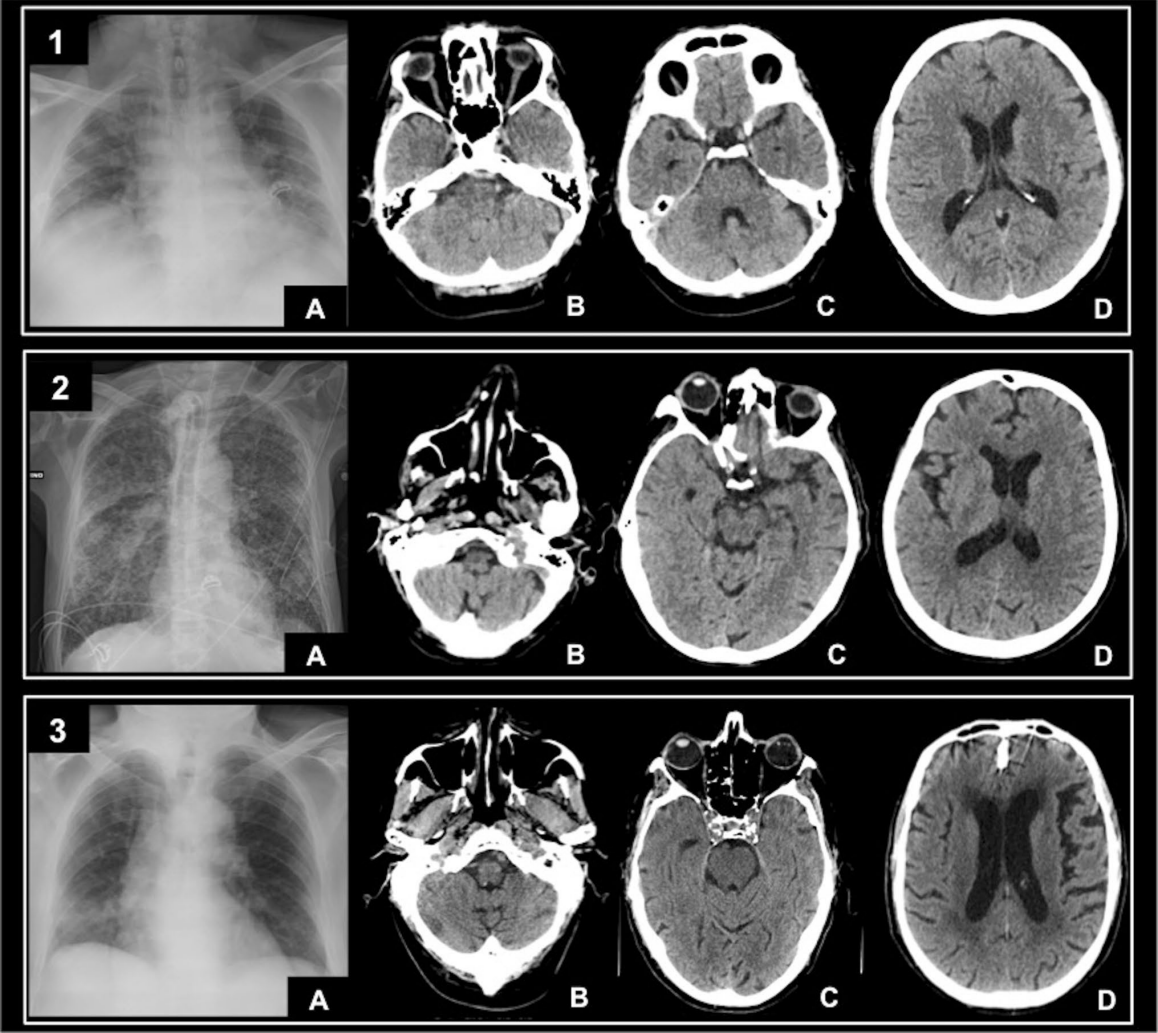
**Panel 1**: Man, 57 years old. The CXR showed advanced lung disease with diffuse consolidations and interstitial involvement: RALE 20 [A]. The brain CT scan was performed for syncope and head trauma in patient with fever. Only the presence of elongation of the vertebral artery (index D, [B]) was found on CT scan by both observers. The CVD-CT score was 0. The patient didn’t need invasive ventilation and survived. **Panel 2**: Man, 69 years old. The CXR showed severe lung disease with diffuse consolidations and interstitial involvement: RALE 30 [A]. The brain CT scan was performed to exclude possible brain lesions in a sedated patient with prolonged mechanical ventilation. Both observers agreed on the presence of elongation of the vertebral artery (index D, [B]) and vessel calcifications of both carotid siphons (index C, [C]). The CVD-CT score was 3. The patient survived. **Panel 3**: Man, 76 years old. The CXR showed moderate lung disease with diffuse consolidations and interstitial involvement: RALE 14 (A). The brain CT scan was performed for disequilibrium in patient with fever. Both observers agreed on the presence of elongation of the vertebral artery (index D, [B]) and vessel calcifications of both carotid siphons (index C, [C]). Moreover, they agreed on the presence of severe leukoaraiosis [D]. The CVD-CT score was 5. The patient needed invasive ventilation but died during hospitalization

After logistic regression only CVD-CT index showed a significant association with mortality. Anyway, since RALE showed an almost significant association (odds ratio 1–1.1, p 0.06), it was included in the regression model. Both forward and backward regressions gave the same results and thus the same odds ratios. The Hosmer–Lemeshow test showed that the regression model adequately fits the data (p = 0.92). Considering the RALE score equal to 0 and 44 (minimum and maximum in the population) the probability of death calculated with the logistic regression model raised from 0.21 to 0.54, with an increase of 0.33. Considering the CVD-CT score equal to 0 and 5 (minimum and maximum in the population) the same probability raised from 0.15 to 0.62, with an increase in 0.47. Therefore, the mortality of the considered patients depends more on the CVD-CT index than on the RALE, probably indicating a condition with a higher impact on mortality risk (Fig. 1). Detailed results of logistic regression analysis are reported in Table 3.

**Table 3:** Odds ratio with 95% confidence interval and* p* values after logistic regression

Variable	*p* value	Odds ratio	95% CI
RALE	0.06	1.05	1.00–1.10
CVD-CT	0.001	1.60	1.20–2.10

*RALE* Radiographic Assessment of Lung Edema score*, CVD-CT* Cerebrovascular disease computed tomography score

## Discussion

To the best of our knowledge, this is the first study investigating a possible relationship between the presence of chronic CVD signs on CT and the risk of fatal outcome in COVID-19 patients. For this purpose, we developed CVD-CT, a rapid visual score based on dichotomous indexes and related both to large and small vessels pathology, which has been found to be significantly associated with increased mortality in our cohort. Moreover, CVD-CT correlated better with risk of death as compared to RALE, whereas other covariates as sex and age, previously reported to be associated with disease severity [15, 16], were not found to be statistically significant on multivariate logistic regression analysis (p 0.36 and 0.48, respectively), despite a higher mortality among males (36% vs 20% among women).

In our group, the mean age (72 ± 14.8 years) was higher as compared to most studies, due to the higher probability of performing a cerebral CT scan for neurological symptoms in elderly patients. In our opinion, this could be the reason of the lack of significant correlation between age and mortality in our study, since several comorbidities associated with increased risk of death in COVID-19 patients are more common in older people.

Among such chronic pathological conditions, cardiovascular disease plays a major role [17]. After the initial involvement of respiratory system, the viral spread can damage many other organs and tissues and trigger a broad spectrum of pathological changes and complex immune responses, whose mechanisms are not yet completely understood [3]. The severe evolution of COVID-19 includes acute respiratory distress syndrome (ARDS) and multi-organ failure, both associated to myocardial dysfunction which is exacerbated by preexisting chronic cardiovascular disorders [3]. Moreover, ACE-2 receptor, the entry point for SARS-CoV-2 to penetrate the target cells, plays a key role on blood pressure regulation. Viral infection downregulates ACE-2 expression, limiting its protective effects on cardiovascular system [18].

In a meta-analysis, both cerebrovascular and cardiovascular diseases were found to be independently associated with increased risk of fatal outcome in COVID-19, without influence by age, gender, respiratory comorbidities, hypertension or diabetes [18]. To explain it, the authors emphasize that both conditions share the same risk factors, often overlapped. Moreover, a link between cerebrovascular and cardiovascular diseases has been found in several studies that demonstrated associations between radiological indicators of one entity with clinical history of the other, and vice versa. The presence of intracranial arterial calcifications has been reported to be predictive of both cerebrovascular and cardiac events [19]. An autopsy study found a significant association of coronary artery ectasia and intracranial artery dolichoectasia in cerebral stroke patients, suggesting a common pathogenesis [20]. Even more important, patients with leukoaraiosis were found to have an almost three-fold risk of death from vascular causes than patients without. Furthermore, they showed a higher risk of dying from a cardiovascular disease than from cerebral stroke itself [21]. The association of WM microangiopathy, age and cardiovascular risk factors, with poor prognoses in patients with COVID-19 has been previously reported [22]: in the absence of a reliable medical history for these patients, microangiopathic changes can serve as a window to the patient’s long-standing underlying risk factors and can provide potentially prognostic insights. With regard to COVID-19 patients, another recent study showed a higher mortality in those with a history of stroke [23].

In our opinion, the presence of CT signs of CVD may be considered as surrogate markers of a systemic disorder affecting the circulatory system. This may explain their association with worse prognosis in COVID-19, where the infective agent penetrates the target cells via a membrane receptor with important regulatory functions over cardiovascular system. The higher predictivity for death of CVD-CT than RALE may be justified by a stronger role of cardiovascular fragility as compared to the severity of pulmonary disease in determining a fatal outcome.

We have several limitations in our study. First of all, it is retrospective and mainly based on imaging signs, with possible biases due to the lack of clinical and anamnestic data (i.e., information about comorbidities such as diabetes or hypertension). Our results have to be confirmed by other studies, investigating the possible influence of clinical, epidemiological or therapeutic factors on prognosis.

Second, the synthetic score that we utilized to summarize neuroimaging signs of CVD in COVID-19 patients was based on CT, since MRI performance is highly problematic in COVID-19 patients, due to isolation and disinfection issues. The patients did not undergo CT angiography, therefore we could not diagnose the possible presence of arterial stenosis. Important indicators of small vessel disease were not considered because not adequately assessed on CT as compared to MRI, such as microbleeds, lacunae and perivascular spaces. The partial assessment of WM microngiopathy represents the major limit of our work.

We also decided to exclude atrophy, although possible on CT, because in our opinion its qualitative evaluation could be strongly influenced by the personal experience on neuroimaging.

Third, our choice to define a simplified CT assessment of chronic CVD carries obvious drawbacks. The surprisingly weak agreement on index C is an example. We think that disagreement was mostly due to the standard window setting used for analysis, which determined misinterpretation of vascular calcifications of the siphon as dural calcifications or partial volume effects from the skull base. As previously suggested in literature, a bone window setting is preferable [8]. Furthermore, we applied a binary method of classification of intracranial calcifications, whereas a quantification method is more appropriate for risk stratification [8]. On the other hand, we point out that our agreement on the diagnosis of leukoaraiosis and dolichoartery was almost optimal, with 98% and 90% of identical classifications, respectively, and the percentage of identical classifications of large vessel disease (LVDsr) was 77%.

Fourth, CVD-CT summarizes imaging signs of large and small vessels disease that, although correlated, have to be considered as separate entities [24]. Our indices C and D may also be related to distinct underlying pathogenetic factors, since dilatative arteriopathies affect the tunica media of the arterial wall and calcified atherosclerotic plaques involve vascular endothelium. The two conditions were found to be associated with different neuroimaging markers of cerebral small vessel disease [24].

Fifth, in a recent work, Kremer et al. [25] described nonconfluent multifocal WM lesions of unclear interpretation as one of the more frequent possible neuroradiological patterns in patients with severe COVID-19. This could represent a bias in our work because such lesions are hard to be differentiated from chronic leukoaraiosis.

Anyway, we aim to test and possibly validate CVD-CT in other groups of selected patients in the future. Lastly, we utilized CXR for radiological assessment of pulmonary disease. As already explained in a previous study from our institution, the RALE score has a good sensitivity and correlates with outcome [12]. Also, in our study, it seems to correlate with risk of mortality. However, although CXR has clear advantages over CT in an emergency setting, due to isolation and disinfection issues, it does not have the same diagnostic power in evaluating COVID-19 patients [26].

## Conclusion

We developed a visual score for a rapid evaluation of indicators of chronic CVD on CT, named CVD-CT, which was associated with an increase of mortality in a cohort of COVID-19 patients. In our opinion, the presence of CT signs of chronic CVD may be related to a condition of fragility of the circulatory system, which constitutes a key risk factor for death in infected patients. Our results need to be confirmed by further studies with larger groups of patients.
